# Impact of Decorative Ceramic Screen Printing on the Optical and Photovoltaic Performance of Glass Covers for BIPV Applications

**DOI:** 10.3390/ma19112420

**Published:** 2026-06-05

**Authors:** Paweł Kwaśnicki, Anna Gronba-Chyła, Dariusz Augustowski, Ludmiła Marszałek, Agnieszka Generowicz, Anna Kochanek, Iga Pietrucha, Krzysztof Barbusiński

**Affiliations:** 1 Research & Development Centre for Photovoltaics, ML System S.A., Zaczernie 190G, 36-062 Zaczernie, Poland; dariusz.augustowski@mlsystem.pl (D.A.); ludmila.marszalek@mlsystem.pl (L.M.); 2 Faculty of Medicine, John Paul II Catholic University of Lublin, Konstantynów 1H, 20-708 Lublin, Poland; amgronba@kul.pl; 3 Department of Physics and Medical Engineering, Rzeszów University of Technology, al. Powstańców Warszawy 6, 35-959 Rzeszów, Poland; 4 Cracow University of Technology, Department of Environmental Technologies, ul. Warszawska 24, 31-155 Kraków, Poland; 5 Cracow University of Technology, Interdisciplinary Center for Circular Economy, Warszawska 24, 31-155 Kraków, Poland; 6 Faculty of Engineering, State University of Applied Sciences in Nowy Sącz, 33-300 Nowy Sącz, Poland; akochanek@ans-ns.edu.pl (A.K.); ipietrucha@ans-ns.edu.pl (I.P.); 7 Department of Water and Wastewater Engineering, Silesian University of Technology, Konarskiego 18, 44-100 Gliwice, Poland; krzysztof.barbusinski@polsl.pl

**Keywords:** BIPV, ceramic screen printing, optical transmittance, photovoltaic efficiency, building integration, decorative solar glass

## Abstract

This study evaluates the effect of decorative ceramic screen printing on the optical and photovoltaic performance of glass covers intended for building-integrated photovoltaics (BIPV). Nine ceramic-printed glass samples with different colors and optical densities were compared with a 4 mm Optiwhite reference glass and a bare silicon solar cell. The samples were characterized by UV-VIS-NIR spectrophotometry, energy-dispersive X-ray spectroscopy (EDS), and electrical measurements under simulated AM 1.5G irradiation at 1000 W/m^2^. The optical results showed that the Optiwhite reference provided the highest transmittance, whereas the printed samples exhibited lower transmission, typically in the range of 60–80% in the visible region, depending on the coating type. Among the decorative variants, sample 1 showed the highest transparency, while sample 6 exhibited the lowest transmittance. The spectral behavior of the coated glasses indicates that the ceramic layers modify the photon flux reaching the solar cell through wavelength-dependent absorption and scattering effects. The photovoltaic measurements confirmed a clear relationship between decorative coating and electrical performance. Relative to the Optiwhite-covered reference cell, the printed samples showed power losses ranging from approximately 17% to 32%, with sample 1 achieving the highest maximum power among the decorative variants at 1.41 W, and sample 4 the lowest at 1.16 W. The main electrical effect of the ceramic coatings was a reduction in short-circuit current, whereas the open-circuit voltage remained nearly constant across the tested samples. EDS analysis identified the presence of ceramic-layer constituents associated with silica-, zinc-, titanium-, iron-, cobalt-, aluminum-, and fluorine-containing compounds, supporting the interpretation of vitrified decorative coatings formed during high-temperature processing. Overall, the results demonstrate that decorative ceramic printing can provide a practical compromise between architectural appearance and photovoltaic output when the optical density of the coating is appropriately controlled.

## 1. Introduction

The dynamic development of renewable energy technologies and the increasingly stringent energy efficiency regulations for buildings—such as the nearly Zero Energy Building (nZEB) standards—pose significant challenges for modern architecture. Building-integrated photovoltaics (BIPV) has emerged as one of the most promising solutions, moving beyond traditional rooftop installations by transforming structural components into active energy generators [1,2].

### 1.1. The Role of BIPV and PV Systems in Modern Architecture

Photovoltaic modules are considered to be building-integrated if they are designed in accordance with the basic requirements for construction works in order to create and/or replace a construction product. After the PV module is dismantled, it must be replaced with an appropriate conventional construction product [3,4]. In this technology, solar cells are regarded as building envelope materials such as tiles, films, modules, or windows. The system maintains the existing specifications of the building envelope materials—such as weather protection, privacy, noise protection, and thermal insulation—while simultaneously generating electrical energy for the building [5,6,7]. BIPV systems constitute an integral part of the building’s exterior envelope, including ventilated facades, skylights, balustrades, and roofing. Unlike conventional PV installations, these modules perform a dual function: technical (providing weather protection, thermal insulation, and acoustic shielding) and energetic [8]. However, aesthetic appeal remains a critical factor for the widespread adoption of BIPV. Traditional, dark-colored crystalline silicon cells often fail to meet the visual requirements of architects, particularly in high-prestige projects or the revitalization of historical sites [9,10]. The growing importance of BIPV in the context of environmental protection also stems from the potential synergy between its energy-related functions and passive functions, such as reducing heat gains in buildings through shading, improving visual comfort, or regulating light transmittance in the case of transparent modules [11]. As a result, BIPV not only generates energy but also contributes to optimising the building’s energy performance at the whole-system level. The authors reviewed BIPV products available on the market and classified them into four subgroups, namely films, tiles, modules, and glazing products incorporating solar cells. They concluded that new PV technologies will lead to more efficient and less expensive BIPV solutions, resulting in shorter payback periods [7,12]. Partial shading is one of the most critical issues concerning power reduction in BIPV systems. It is difficult to avoid partial shading effects throughout the year due to surrounding obstacles near BIPV installations [13,14]. The methodology for assessing environmental comfort in BIPV evaluates aspects such as thermal comfort and visual comfort. Visual comfort is a parameter that must be taken into account, as the examined photovoltaic devices can significantly alter light perception; they can provide appropriate solar shading to reduce glare and prevent undesirable overheating effects, which are also related to thermal comfort in indoor spaces [15,16].

### 1.2. Application of Ceramic-Printed Glass

Studies are available that describe the manufacturing process of coloured photovoltaic modules using various technologies. Experimental assessments of the electrical performance of coloured modules have also been carried out, including under real exposure conditions [17,18,19,20,21]. The challenge of low visual attractiveness is addressed through the application of decorative ceramic screen printing on glass substrates. Coloring the front glass of photovoltaic (PV) modules using digital ceramic printing helps to conceal PV modules when they are integrated into existing building façades as building-integrated photovoltaics (BIPV), while still allowing sufficient light to pass through for electricity generation [22,23]. The coloring technology may involve pigments, interference coatings, or fluorescent dyes [24]. This technology allows photovoltaic modules to adopt almost any color or texture, effectively masking the solar cells while maintaining adequate light transmittance [25]. Ceramic inks, fused into the glass structure during the tempering process (650–720 °C), offer several key advantages: **Superior Durability**: High resistance to UV radiation, mechanical abrasion, and aggressive chemical agents. **Optical Parameter Control**: Precise engineering of light transmittance (LT), which directly impacts the balance between aesthetic camouflage and the electrical yield of the cells. **Structural Safety**: The thermal treatment required for the ceramic print imparts tempered or semi-tempered properties to the glass, which is essential for facade safety and structural integrity [26,27,28]. Research has also been conducted on the development of ceramic BIPV modules by depositing a thin layer of amorphous silicon onto a ceramic substrate [29]. To meet various BIPV specifications, different types of large-area substrates can be used for thin-film photovoltaic modules, such as glass, plastic, ceramics, graphite, or metal [30,31].

### 1.3. Benefits and Market Demand

The global BIPV market is characterized by exceptional growth dynamics. Projections indicate that its market value, estimated at approximately USD 28 billion in 2024, is expected to exceed USD 130 billion by 2026, representing a Compound Annual Growth Rate (CAGR) of over 20%. The primary drivers of this trend include: **Sustainable Development:** The reduction in the carbon footprint of buildings and the pursuit of energy self-sufficiency in dense urban environments where roof space is limited [32]. **Economic Scalability:** Replacing traditional cladding materials (such as stone or composites) with BIPV modules allows for a partial return on investment in construction materials through on-site electricity generation [33,34,35]. **Design Flexibility:** The availability of a wide palette of colors (e.g., Dark Grey, Anthracite, Gold) enables the harmonious integration of solar panels into the architectural environment, opening the market to commercial and public sector investments [36,37,38,39,40]. The lifespan of BIPV panels is estimated at 30 years [41], although some sources report the possibility of extending it to 50 years [42]. These panels can be installed on both existing buildings and renovated structures [41,42]. The potential to achieve net-zero energy buildings [43,44], through the use of various façades and building orientations to distribute energy production throughout the day [45,46], as well as the system’s contribution to improving the energy efficiency of building envelopes, are among the advantages of BIPV façades [47,48,49].

Currently, the scientific literature lacks a comprehensive review of models and simulation frameworks used to assess and investigate the relationship between colors and performance in BIPV.

The aim of this study is to experimentally assess how decorative ceramic screen printing on glass affects optical transmittance and the photovoltaic response of silicon solar cells in a BIPV-relevant configuration. The work focuses on the trade-off between aesthetic modification and electrical performance and does not include a full environmental or life-cycle assessment.

The novelty of this study lies in the combined experimental assessment of industrially prepared ceramic-printed decorative glass used as BIPV cover glazing, considering optical, compositional, and photovoltaic aspects within one consistent comparative framework. Unlike studies focused mainly on architectural appearance or general coloured-BIPV concepts, the present work directly compares nine decorative glass variants under identical measurement conditions and relates their wavelength-dependent transmittance to the electrical response of the same monocrystalline silicon solar cell used as a constant comparative detector. In this way, the study provides practical experimental evidence for the trade-off between aesthetic modification and photovoltaic performance in façade-oriented BIPV glazing.

## 2. Experimental Methodology

The experimental procedure was designed to evaluate the optical, compositional, and electrical effects of decorative ceramic coatings deposited on glass substrates intended for BIPV cover applications.

### 2.1. Analytical Instruments

#### 2.1.1. Optical Characterization

The spectral transmittance of the decorative glass samples was measured using a Jasco V-670 UV-VIS-NIR spectrophotometer (JASCO International Co., Ltd., Tokyo, Japan). Measurements were performed over the wavelength range from 250 to 2500 nm in order to assess the spectral transmission behavior of the coatings in the ultraviolet, visible, and near-infrared regions.

Particular attention was given to the visible and near-infrared ranges, because these spectral regions determine both the apparent visual character of the glass and the effective photon flux reaching the silicon solar cell. The Optiwhite glass served as the high-transmittance reference for all optical comparisons.

Colorimetric characterization was additionally performed using the Color Analysis module implemented in the JASCO Spectra Manager software (version 2.09.03) The analysis was carried out under the D65 illuminant, and the results were expressed in the CIE XYZ color space together with the corresponding chromaticity coordinates x,y. The purpose of this measurement was to provide an objective numerical description of the visual appearance of the selected printed glass surfaces and to complement the spectral transmittance analysis.

The transmittance spectra were recorded in the broad 250–2500 nm range in order to provide complete optical characterization of the decorative glass, including the ultraviolet, visible, and near-infrared regions. However, for photovoltaic interpretation related to the silicon solar cell, the most relevant spectral interval is 250–1200 nm. Therefore, the current-related discussion and the AM1.5G-weighted analysis presented in this work were restricted to this wavelength range, while the wider spectral window was retained for completeness of the optical characterization of the BIPV cover glass.

#### 2.1.2. Elemental Characterization

Elemental analysis of the decorative coatings was performed by energy-dispersive X-ray spectroscopy using an Oxford Instruments Swift ED3000 system (Oxford Instruments, Abingdon, UK). The measurements were carried out under vacuum at an accelerating voltage of 15 kV.

The purpose of the EDS analysis was to identify the principal inorganic constituents of the ceramic layers, including frit-related components and pigment-related elements, and to compare them with the unprinted glass reference. In the revised manuscript, the EDS data should be presented as semi-quantitative elemental contents, expressed as atomic or weight percentages, in order to support the compositional interpretation more rigorously. The EDS analysis performed in this work was intended as a semi-quantitative elemental characterization of the decorative ceramic coatings and the corresponding glass reference. The method provides information on the presence and relative abundance of elements, but it does not by itself enable definitive identification of chemical phases, oxidation states, or spatial compositional heterogeneity within the coating. Therefore, the EDS results are interpreted here as supportive compositional data rather than as phase-specific chemical proof.

#### 2.1.3. Photovoltaic Measurements

To determine the effect of decorative glass on photovoltaic response, each sample was placed above a standard silicon solar cell in a second-surface configuration, meaning that the printed side of the glass faced the cell and the incident light first passed through the unprinted glass surface. This arrangement was selected to mimic a realistic cover-glass geometry relevant for BIPV module integration. The measurements were carried out using a solar simulator equipped with a Xenon 1000W, short arc (4 mm) lamp of 33000 luminous flux (Ushio Inc., Tokyo, Japan), at a controlled cell temperature of 25 °C.

The electrical measurements were performed under simulated AM1.5G illumination at an irradiance of 1000 Wm^−2^. The photovoltaic reference device was a monocrystalline SunPower Maxeon interdigitated back-contact silicon solar cell (SunPower Corporation, San Jose, CA, USA) with an active area of approximately 153 cm^2^, used throughout the study as a constant comparative detector. The investigated glass samples were placed above the cell in a second-surface configuration, and the measured quantities included $P_{max}$, $I_{SC}$, $V_{OC}$, and fill factor. For comparative purposes, the measured short-circuit current may also be expressed as current density according to $J_{SC}=I_{SC}/A$, where A is the active cell area. The present measurements were intended for relative comparison of glass-induced optical losses under identical laboratory conditions rather than for IEC-certified photovoltaic device characterization.

The relative performance loss was determined from the ratio of the measured maximum power of the decorated configuration to that of the Optiwhite-covered reference cell. This comparison enabled direct assessment of the electrical penalty associated with the decorative ceramic print under otherwise identical irradiation conditions.

In order to relate the measured optical transmission of the cover glasses to the current response of the silicon solar cell, an AM1.5G-weighted transmittance factor was additionally calculated from the measured spectra. For each sample, the weighted transmittance in the 250–1200 nm range was defined as

$$T_{w}=\frac{\int_{250}^{1200}T(\lambda )\;S_{AM1.5G}(\lambda )\;d\lambda }{\int_{250}^{1200}S_{AM1.5G}(\lambda )\;d\lambda }$$

where T(λ) is the measured spectral transmittance of the glass and $S_{AM1.5G}(\lambda )$ is the reference AM1.5G solar spectrum. The obtained parameter was treated as a relative optical indicator of the fraction of incident solar radiation transmitted through the decorative glass toward the cell. To facilitate comparison with the photovoltaic measurements, the weighted transmittance was further normalized to the Optiwhite reference:

$$R_{opt}=\frac{T_{w,sample}}{T_{w,Optiwhite}}$$

and compared with the experimentally measured short-circuit current ratio

$$R_{Isc}=\frac{I_{SC,sample}}{I_{SC,Optiwhite}}.$$

Because the same monocrystalline silicon solar cell was used in all measurements, this procedure provides a consistent basis for evaluating relative current-related losses induced by the investigated decorative glass covers. A rigorous absolute prediction of $J_{SC}$ would additionally require the spectral response of the solar cell, e.g., EQE(λ).

### 2.2. Material Selection and Characterization

The study included nine decorative ceramic-printed glass samples, denoted as samples 1–9, and two reference configurations. The transparent reference substrate was 4 mm thick Optiwhite glass, which served as the baseline material for optical and photovoltaic comparison. For tested samples float tempered glasses (front and back) with a 4 mm thickness with a trapezoidal edge, EVA (Ethylene–vinyl acetate) and lamination film with a thickness of 0.38 mm were used. The photovoltaic reference device used in this study was a monocrystalline silicon SunPower/Maxeon interdigitated back-contact (IBC) solar cell with an all-back-contact architecture. According to the manufacturer’s technical documentation, this cell family is based on a monocrystalline silicon structure with tin-coated copper rear metallization, a thickness of approximately 150 μm, and an active area of about 153 cm^2^. It should be emphasized that, in the present work, the cell was used as a constant comparative detector for assessing the influence of decorative glass covers under identical experimental conditions, rather than for determining its certified photovoltaic efficiency. Therefore, the discussion focuses mainly on relative changes in $I_{SC}$ and $P_{max}$, while the absolute FF values are influenced by the non-optimized laboratory contacting and measurement configuratio. An additional electrical reference was provided by a bare silicon solar cell measured without any glass cover.

The investigated samples differed in visual appearance, color tone, and effective transparency, representing commercially relevant decorative variants for façade-oriented BIPV applications. The ceramic layers were applied to the glass substrates by screen printing, followed by thermal processing typical of architectural glass decoration and strengthening. The ink used for the process are commercially available material from TecGlass C.O. containing, according to the producer Frit (zinc, quartz and silica), inorganic pigments (iron oxide, cobalt, and TiO_2_) and the medium.

### 2.3. Sample Preparation and Processing

After deposition of the ceramic pattern, the samples were subjected to a two-stage thermal treatment consisting of drying at 110 °C and subsequent firing/tempering in the range of 650–720 °C. This treatment ensured fusion of the ceramic layer with the glass surface and produced glass specimens with properties characteristic of tempered or heat-strengthened architectural glass.

To assess the thermal history and structural character of the specimens, fracture behavior was qualitatively examined after breaking selected samples. Based on the observed fragmentation patterns, the tested glass panes were classified as either fully tempered or heat-strengthened, depending on the crack morphology and fragment size distribution.

For spectroscopic and elemental characterization, smaller pieces were cut from the original decorative glass panes. Unprinted glass fragments were also prepared as a reference for the EDS analysis in order to distinguish substrate-related elements from those introduced by the ceramic coating.

Because the samples originated from industrial decorative glass processing, the exact trade names and full proprietary formulations of the ceramic inks are not disclosed. Nevertheless, all accessible parameters relevant to experimental reproducibility, including substrate type, thickness, process temperatures, analytical methods, and optical-electrical test configuration, are provided in this work.

**Figure 1:**
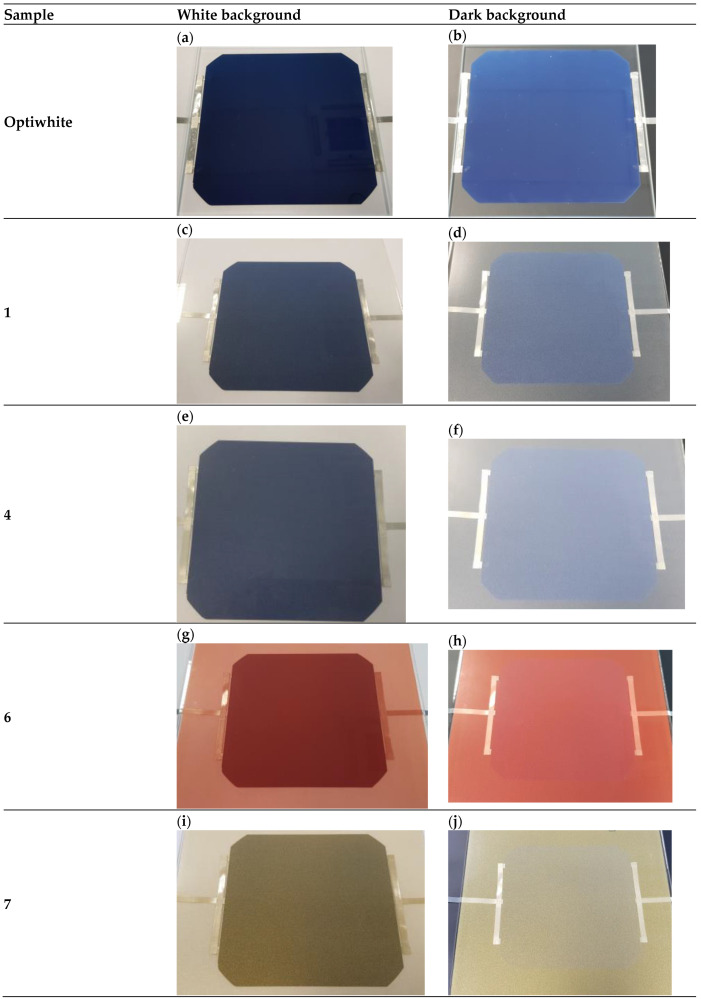
Photographs of the prepared samples under white and dark backgrounds: (**a**) Optiwhite (white background); (**b**) Optiwhite (dark background); (**c**) Sample 1 (white background); (**d**) Sample 1 (dark background); (**e**) Sample 4 (white background); (**f**) Sample 4 (dark background); (**g**) Sample 6 (white background); (**h**) Sample 6 (dark background); (**i**) Sample 7 (white background); (**j**) Sample 7 (dark background).

Example of the samples are presented in Figure 1a–j.

For the electrical performance analysis, the glass samples were positioned in a “second surface” configuration relative to the solar cell (i.e., the printed layer facing the cell), ensuring that incident light first passed through the unprinted side of the glass.

For the subsequent spectroscopic and elemental analyses, smaller specimens were extracted from the original large-format glass panes. Unprinted glass fragments were also prepared to serve as a baseline for the elemental composition analysis, allowing for the isolation of specific elements introduced by the ceramic printing process.

The present study was designed as a comparative assessment of decorative BIPV cover glasses under one fixed experimental configuration, using the same monocrystalline silicon solar cell as a constant comparative detector. Therefore, the discussion focuses mainly on relative differences in transmittance, $I_{SC}$, and maximum power measured under identical conditions. In addition, the study did not include direct microstructural characterization of the decorative ceramic layer by SEM, cross-sectional SEM, AFM, or TEM. Consequently, parameters such as coating morphology, local thickness homogeneity, porosity, and pigment dispersion were not directly resolved and are not treated here as experimentally verified microstructural descriptors. The lack of repeated measurements and formal statistical error analysis should also be regarded as a limitation of the present work. Future studies should therefore include position-dependent repetition protocols with reporting of mean values and standard deviations, together with direct microscopic characterization, in order to establish more rigorous correlations between coating microstructure, optical behavior, and photovoltaic response.

## 3. Results and Discussion

### 3.1. Optical Transmittance

**Figure 2:**
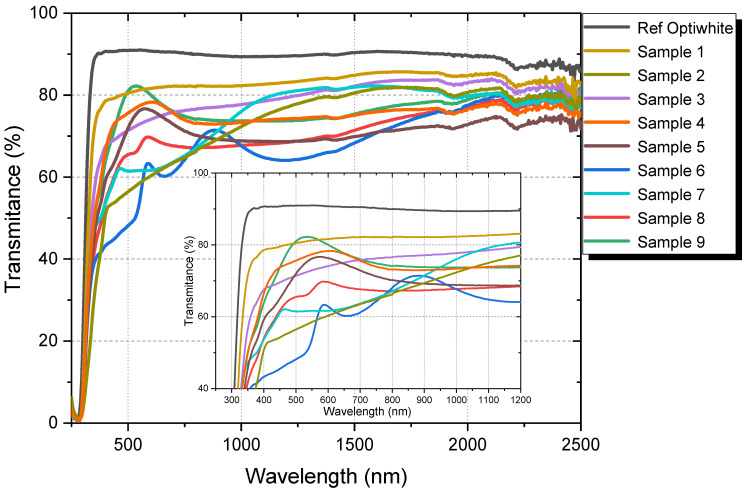
UV-VIS-NIR transmittance spectra of ceramic-printed glass samples compared with the non-printed Optiwhite reference, measured in the 250–2500 nm range and zoom on region 250–1200 nm. The full spectral range is shown for complete optical characterization of the decorative glass, whereas the photovoltaic interpretation for the silicon solar cell and the AM1.5G-weighted analysis are focused on the 250–1200 nm interval.

The transmittance spectra of all samples were measured in the UV-VIS-NIR range and compared with the 4 mm Optiwhite reference glass (see Figure 2). Although Figure 2 presents the full transmittance spectra in the 250–2500 nm range, the photovoltaic relevance for the silicon solar cell is concentrated in the 250–1200 nm interval. For this reason, the qualitative discussion of Figure 2 is complemented by a current-oriented analysis based on the AM1.5G-weighted transmittance calculated only over 250–1200 nm. The extended spectral range is nevertheless useful for describing the overall optical behavior of the decorative glass, including UV attenuation and near-infrared transmission relevant to glazing characterization. As expected, the reference glass exhibited the highest and most stable transmittance over the visible and near-infrared regions, reaching approximately 90% in the main transparent range. This behavior confirms the suitability of the low-iron substrate as a benchmark for evaluating decorative cover-glass losses in BIPV applications. The introduction of decorative ceramic layers led to a clear reduction in optical transmission in the visible range. For most printed samples, the transmittance remained within approximately 60–80%, although the detailed spectral profiles depended on the color and composition of the coating. Among the tested variants, sample 1 showed the highest transparency, whereas sample 6 exhibited the lowest transmittance, indicating the strongest optical attenuation among the decorative coatings.

The spectral curves also showed local minima that can be associated with selective absorption or scattering introduced by the pigment-containing ceramic layers. In particular, the more strongly attenuated samples, such as sample 4 and sample 6, displayed deeper spectral depressions than the more transparent variants. This behavior is consistent with the use of pigment systems that alter the visual appearance of the glass by modifying spectral transmission rather than by acting as a neutral optical filter.

In the near-infrared range, several decorative samples retained comparatively higher transmittance than in the visible region. This observation is relevant from an optical point of view because silicon solar cells remain photoactive over part of the visible-to-near-infrared range up to the silicon band-edge region. However, in the absence of direct spectral responsivity or EQE(λ) data for the cell used in this study, these results should not be interpreted as rigorous experimental proof of preserved NIR harvesting efficiency. Instead, the near-infrared transmittance behavior is discussed here only as an optical trend that may contribute to the comparatively smaller current losses observed for some glass variants under identical measurement conditions. A marked drop in transmittance below approximately 380 nm was observed for all samples, including the reference glass, and the ceramic coatings further enhanced this attenuation in the ultraviolet range. Although such behavior may be beneficial for limiting UV exposure of module components, the present work did not include a dedicated durability study, and therefore, no direct conclusion on long-term protective performance is drawn. Possible micro-scale shadowing, local optical non-uniformity, and thermal effects are considered here only as physically plausible interpretation pathways inferred from the optical and photovoltaic trends, and they were not directly verified by dedicated microscopic imaging or thermal-mapping measurements. Their confirmation requires additional microscopy and thermographic experiments. Overall, the optical results show that decorative ceramic printing provides substantial control over appearance, but this control is accompanied by measurable spectral losses relevant to photovoltaic performance.

### 3.2. Colorimetric Characterization

To supplement the visual description of the decorative coatings, selected printed glass surfaces were additionally characterized in the CIE color space using the Jasco Color Analysis module under D65 illumination conditions. The measured parameters were expressed in the CIE XYZ system together with the chromaticity coordinates x,y, which provide an objective numerical description of the observed colors.

The obtained results (see Table 1) confirmed clear differences between the analyzed colored regions, indicating that the decorative ceramic prints can be differentiated not only visually but also by quantitative chromaticity data. In particular, the measured x,y coordinates showed distinct distributions for blue, red, green, yellow, black, and white printed regions, confirming that the ceramic printing process produces reproducible color states that can be described numerically in a standardized colorimetric framework.

These data support the interpretation that the decorative effect of the coatings arises from wavelength-dependent modification of transmitted and reflected radiation, which is consistent with the spectral behavior observed in the transmittance measurements. Therefore, the colorimetric analysis provides complementary information to the UV-VIS-NIR results and strengthens the materials-based description of the decorative BIPV glass.

At the same time, it should be noted that the currently available colorimetric dataset refers to selected measured samples or printed regions and does not constitute a complete standardized color database for all decorative variants investigated in the photovoltaic part of this study. For this reason, the colorimetric results are presented here as supportive characterization data and are interpreted separately from the experimentally verified transmittance and photovoltaic measurements.

### 3.3. Photovoltaic Performance

The photovoltaic measurements showed that the ceramic-printed covers directly affected the electrical output of the silicon solar cells. The bare cell delivered the highest maximum power at 1.80 W, while the cell covered with reference Optiwhite glass reached 1.70 W. All decorative glass variants produced lower power values than the Optiwhite reference, confirming that the ceramic layers introduced additional optical losses.

Among the printed samples, sample 1 exhibited the best photovoltaic performance, with a maximum power of 1.41 W and a power ratio of 0.83 relative to the Optiwhite reference. In contrast, sample 4 showed the lowest performance, with a maximum power of 1.16 W and a power ratio of 0.68, corresponding to an approximately 32% power loss. The remaining decorative variants produced intermediate results, typically corresponding to power losses in the range of about 20–28%.

Detailed photovoltaic parameters, including short-circuit current $I_{SC}$, open-circuit voltage $V_{OC}$ and fill factor, are summarized in Table 2. In line with the non-optimized laboratory contacting configuration, the absolute FF values are lower than typical commercial cell values and should be interpreted as measurement-setup-related, rather than as intrinsic device limitations.

The measured electrical parameters indicate that the dominant effect of the decorative ceramic layers was a reduction in short-circuit current. The $I_{SC}$ values of the printed samples ranged from 3.97 A to 5.32 A, compared with 5.71 A for the Optiwhite-covered reference. By contrast, the open-circuit voltage remained relatively stable for all tested variants, with values close to 0.65 V, which indicates that the ceramic glass acted mainly as an optical modifier rather than as a factor altering the intrinsic semiconductor properties of the cell.

The strongest photovoltaic performance was observed for the sample with the highest optical transmittance, and the weakest performance was obtained for one of the most optically attenuating coatings. This agreement between optical and electrical measurements supports the conclusion that the reduction in photon flux reaching the cell was the principal reason for the decrease in $P_{max}$. In other words, the experimental dataset shows a consistent trade-off between decorative appearance and photovoltaic output.

The fill factor values showed some variation among the printed samples, ranging from about 36% to 50%, which is consistent with the non-optimized laboratory contacting configuration used in this comparative setup. Because of this configuration-related limitation, FF is not treated as a primary figure of merit in this work; instead, the analysis focuses mainly on the robust and directly supported correlations between spectral transmittance, short-circuit current and their normalized ratios versus the Optiwhite reference.

**Figure 3:**
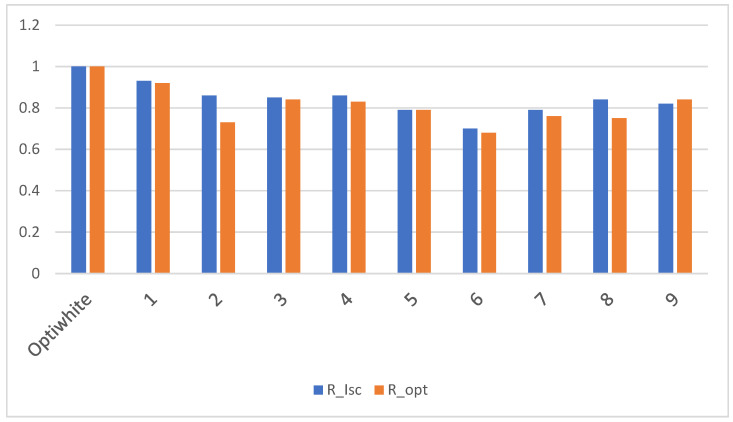
Comparison of the normalized AM1.5G-weighted transmittance $R_{opt}$ and normalized short-circuit current $R_{I_{SC}}$ for the decorative glass samples and the Optiwhite reference.

The photovoltaic analysis presented in this work was intended as a comparative assessment of the immediate electrical response of the same silicon solar cell measured under identical AM1.5G laboratory conditions with different decorative glass covers. To quantify the correspondence between optical and electrical response, the AM1.5G-weighted transmittance in the 250–1200 nm range was normalized to the Optiwhite reference, yielding the factor $R_{opt}=T_{w,sample}/T_{w,Optiwhite}$ with $T_{w,Optiwhite}=89\%$, and compared with the normalized short-circuit current $R_{I_{SC}}=I_{SC,sample}/I_{SC,Optiwhite}$ listed in Table 2. For all decorative variants, $R_{opt}$ and $R_{I_{SC}}$ lie in comparable ranges (approximately 0.68–0.92) and follow very similar trends, confirming that the reduction of transmitted photon flux is the dominant mechanism governing the current loss. In particular, sample 6, which exhibits one of the lowest weighted transmittance ratios ($R_{opt}\approx 0.68$), also shows the strongest current reduction ($R_{I_{SC}}\approx 0.70$), whereas samples with higher $R_{opt}$, such as samples 1 and 9, maintain correspondingly higher $R_{I_{SC}}$. The small residual differences between $R_{opt}$ and $R_{I_{SC}}$ can be attributed to the use of the standard AM1.5G spectrum without convolution with the device-specific spectral responsivity, as well as to experimental uncertainties of the optical and electrical measurements. The close agreement between $R_{opt}$ and $R_{I_{SC}}$ across all samples, as shown in Figure 3, illustrates that the sample-to-sample variations in short-circuit current are largely governed by the AM1.5G-weighted transmittance of the decorative glass, with only minor discrepancies attributable to the lack of convolution with the device-specific EQE and experimental uncertainties. Accordingly, the study focuses on the relative changes in $P_{m}$, $I_{SC}$, $V_{OC}$, and FF induced by the cover-glass variants, rather than on full device diagnostics or module qualification. Advanced characterization methods such as full J-V curve analysis, EQE/IPCE spectroscopy, series and shunt resistance extraction, temperature-dependent measurements, and long-term stability or thermal-cycling tests were outside the scope of the present study and are required for complete validation of practical module reliability.

Additionally, to further verify the optical origin of the observed current losses, the measured transmittance spectra were weighted with the AM1.5G solar spectrum and integrated in the 250–1200 nm range. The resulting normalized weighted transmittance factor $R_{opt}$ followed the same trend as the experimentally measured short-circuit current ratio $R_{Isc}$, confirming that the dominant effect of the decorative ceramic coatings was the reduction of the transmitted photon flux reaching the silicon cell. In particular, the samples with higher optical transparency exhibited higher $I_{SC}$, whereas the more strongly attenuating coatings led to lower current values. This agreement supports the interpretation that the decrease in photovoltaic output was governed primarily by spectral transmission losses introduced by the decorative glass rather than by changes in the intrinsic electrical properties of the cell. It should be noted that the AM1.5G-weighted transmittance is used here as a comparative optical metric; a full physical estimation of the short-circuit current density would require convolution of the transmitted spectrum with the spectral response of the device.

The expanded analysis shows that the main photovoltaic penalty introduced by the decorative ceramic coatings is associated with current loss rather than voltage loss. This interpretation is supported by the experimental data, in which $I_{SC}$ decreases markedly across the decorative variants, whereas $V_{OC}$ remains relatively stable, indicating that the coatings primarily modify the spectral photon flux reaching the silicon cell. When considered together with the transmittance results and the EDS-derived compositional information, the dataset provides a materials-oriented basis for selecting decorative BIPV glass with an improved balance between façade aesthetics and electrical output.

The present interpretation is based on comparative experimental correlations between spectral transmittance, semi-quantitative compositional data, and photovoltaic response measured under identical illumination conditions. No optical simulations such as FDTD, ray tracing, or transfer-matrix modeling were performed, and no carrier-transport or device-resolved EQE analysis was available in the present work. Therefore, absorption-, scattering-, and reflectance-related losses are discussed here only as plausible physical contributors to the observed attenuation of transmitted radiation and the resulting decrease in $I_{SC}$ and power, rather than as quantitatively separated mechanisms. A more rigorous mechanistic decomposition of photon-loss pathways will require future simulation-supported and EQE-resolved studies.

### 3.4. Elemental Composition

The semi-quantitative EDS results indicate that the analyzed decorative coatings share a common glass–ceramic matrix dominated by O, Si, Na, and Zn, with oxygen ranging from 46.5 to 47.9 wt.%, silicon from 17.0 to 21.4 wt.%, sodium from 10.1 to 11.4 wt.%, and zinc from 13.8 to 16.2 wt.% across the reported samples. This compositional consistency suggests that the decorative variants were based on a similar frit-containing formulation, whereas the differences in appearance and photovoltaic response were associated mainly with variations in minor additives and pigment-related elemental contributions. The high contents of Si and O are consistent with a silica-rich vitrified matrix, while the substantial Zn contribution supports the presence of zinc-containing frit components that may promote coating fusion and adhesion during firing. The relatively stable Na content across all samples further suggests compatibility with soda-lime architectural glass processing, whereas minor amounts of Al, F, Ti, and Zr indicate the presence of additional formulation modifiers that may influence durability, processability, and optical behavior. Table 3 presents the semi-quantitative EDS composition of selected ceramic-printed glass coatings in wt.% and at.%, and Table 4 summarizes the principal elemental constituents together with their expected functional relevance to optical and photovoltaic performance. This combined presentation enables a comparative interpretation of the relationship between elemental composition, transmittance behavior, and the corresponding changes in photovoltaic output discussed in Section 3.1 and Section 3.2. However, it should be emphasized that the EDS analysis in the present work provides elemental information only and does not, by itself, allow definitive identification of chemical phases, oxidation states, or spatial compositional heterogeneity within the coating. Therefore, Fe-, Co-, Ti-, Zn-, Al-, F-, and Si-containing contributions are interpreted here as semi-quantitative compositional indicators rather than as directly confirmed pigment phases or phase-pure compounds. In the absence of elemental mapping or complementary phase-sensitive techniques such as XPS, XRD, Raman, or FTIR, the EDS results should be regarded as supportive compositional evidence rather than definitive chemical proof.

Among the analyzed coatings, sample 7 showed the highest Si content at 21.4 wt.% and 16.5 at.%, whereas sample 4 exhibited the lowest Si content at 17.0 wt.% and 12.0 at.%. Sample 4 also showed the lowest Zn content at 13.8 wt.% and the highest carbon contribution at 9.5 wt.% and 15.7 at.%, which distinguishes it from the more frit-dominated coatings. By contrast, samples 1 and 5 were compositionally very similar, each containing 15.6 wt.% Zn and about 20 wt.% Si, which suggests that relatively small changes in the minor constituents may still produce measurable differences in optical and photovoltaic behavior.

Titanium was present in all reported coatings at low levels, ranging from 0.5 wt.% in sample 6 to 1.3 wt.% in sample 2. Although these concentrations are modest, Ti-containing compounds are commonly associated with scattering and opacity control in ceramic systems, so even small differences in Ti content may contribute to the observed variation in visible transmittance. Fluorine ranged from 0.9 wt.% in sample 5 to 2.3 wt.% in sample 7, while aluminum remained a minor constituent between 0.3 and 0.7 wt.%.

A notable difference was observed for sample 6, in which Fe was detected at 0.4 wt.% and 0.1 at.%. This result suggests a distinct pigment-related contribution in that coating and is consistent with the lower optical transmittance reported for this sample. Potassium was detected only in samples 2, 4, 6, and 7 at 0.2 wt.%, indicating an additional minor compositional variation within the decorative set.

When considered together with the optical and photovoltaic results, the EDS data indicate that the best-performing decorative coating, sample 1, was not defined by a unique major-element composition, but rather by a favorable balance within the common frit-based matrix. In contrast, sample 4, which showed the lowest maximum power output, was distinguished by lower Si and Zn contents and a markedly higher carbon contribution, consistent with its stronger optical attenuation. Overall, the EDS results support the interpretation that the decorative layers are vitrified ceramic coatings based on a shared silicate–zinc matrix, with sample-to-sample differences introduced mainly by minor additives and pigment-related components that influence optical transmission and, consequently, photovoltaic response.

Accordingly, terms such as coating uniformity, local scattering heterogeneity, or micro-shading are used in this work only in an interpretative sense, based on indirect correlations between spectral transmittance, semi-quantitative elemental composition, and photovoltaic response, and not as conclusions derived from direct microscopic evidence.

### 3.5. Qualitative Fracture Behavior

Selected samples were subjected to a qualitative post-fracture inspection after manual edge fracture in order to assess the fragmentation behavior of the thermally processed glass. The observed fracture patterns were consistent with the expected behavior of thermally strengthened architectural glass and were used only for qualitative classification of the samples as fully tempered or heat-strengthened, depending on fragment size and crack morphology. No evidence of coating delamination prior to fracture was observed during specimen handling, indicating that the ceramic decoration remained integrated with the glass surface after firing. Because this procedure was not performed as a standardized mechanical test, the results should be interpreted as supportive structural observations rather than as a quantitative measure of mechanical strength or integrity.

## 4. Conclusions and Summary

This study experimentally evaluated the influence of decorative ceramic screen printing on the optical transmittance and photovoltaic performance of glass covers intended for BIPV applications, using one constant monocrystalline silicon solar cell configuration under identical AM1.5G laboratory conditions. The results showed that the ceramic layer acts primarily as an optical modifier that reduces spectral transmittance and, consequently, the short-circuit current and maximum power of the underlying cell, while leaving the open-circuit voltage almost unchanged.

Relative to the low-iron Optiwhite reference glass, which provided the highest transmittance and best electrical response, the decorative coatings introduced power losses of approximately 17–32%, depending on their effective optical attenuation. Within the decorative set, sample 1 combined the highest optical transparency with the most favorable photovoltaic performance (1.41 W, ≈17% loss), whereas sample 4 exhibited the strongest attenuation and the largest power reduction (1.16 W, ≈32% loss).

The decrease in maximum power closely followed both the measured transmittance spectra and the AM1.5G-weighted transmittance, confirming that current loss driven by spectral attenuation is the dominant penalty mechanism for the investigated ceramic coatings. Semi-quantitative EDS analysis indicated that all decorative layers share a broadly similar silicate–zinc glass–ceramic matrix with pigment-related additives, and that variations in optical density within this common formulation govern the balance between façade aesthetics and photovoltaic output.

Overall, the comparative dataset delivered in this work links wavelength-dependent optical losses in decorative BIPV glass directly to the corresponding electrical penalty under identical measurement conditions, demonstrating that industrial decorative ceramic printing can offer a practical compromise between architectural appearance and photovoltaic performance when the optical density of the coating is carefully controlled.

## Figures and Tables

**Table 1:** CIE 1931 XYZ color coordinates and chromaticity coordinates x,y for selected printed glass regions measured under D65 illumination.

Sample	X	Y	I
ref	0.221	0.243	1 × 10^3^
1	0.254	0.272	1 × 10^3^
4	0.269	0.291	1 × 10^3^
6	0.55	0.312	1 × 10^3^
7	0.510	0.466	1 × 10^3^

**Table 2:** Photovoltaic parameters of the measured samples under AM1.5G illumination, including normalized AM1.5G-weighted transmittance $R_{opt}$ and normalized short-circuit current $R_{I_{SC}}$ relative to the Optiwhite reference.

Sample ID	Sample Description/Color	Pm [W]	Isc [A]	Voc [V]	FF [%]	Power Ratio (vs. Optiwhite)	R_Isc_	T_w_ [%]	R_opt_
**Reference**	No Glass (Bare Cell)	1.80	6.29	0.648	44.15	1.06	-		
**Reference**	Optiwhite Glass	1.70	5.71	0.659	45.24	1.00	1.00	1	-
**1**	Dark Grey	1.41	5.32	0.659	40.19	0.83	0.93	81.48	0.92
**2**	Silver	1.34	4.88	0.657	41.87	0.79	0.86	64.57	0.73
**3**	Grey	1.25	4.86	0.655	39.42	0.74	0.85	74.97	0.84
**4**	RGB Grey	1.16	4.93	0.657	35.81	0.68	0.86	73.95	0.83
**5**	Graphite	1.23	4.50	0.657	41.59	0.72	0.79	69.92	0.79
**6**	Brown	1.28	3.97	0.651	49.56	0.76	0.70	60.60	0.68
**7**	Gold	1.23	4.49	0.654	41.97	0.72	0.79	67.34	0.76
**8**	Metallic	1.23	4.80	0.658	39.04	0.72	0.84	66.51	0.75
**9**	Light Grey	1.24	4.67	0.654	40.59	0.73	0.82	75.09	0.84

**Table 3:** EDS-derived semi-quantitative elemental composition of selected decorative ceramic coatings on glass (wt.% and at.%).

1			2			4		
Element	%weight	%atom.	Element	%weight	%atom.	Element	%weight	%atom.
C	1.9	3.4	C	2.6	4.5	C	9.5	15.7
O	47.5	63.4	O	47.7	63.1	O	46.5	57.6
F	1	1.1	F	1.5	1.7	F	1	1
Na	11.4	10.6	Na	10.7	9.9	Na	10.1	8.7
Al	0.3	0.2	Al	0.7	0.6	Al	0.3	0.2
Si	20.6	15.7	Si	19.4	14.6	Si	17	12
Ti	0.6	0.3	K	0.2	0.1	K	0.2	0.1
Zn	15.6	5.1	Ti	1.3	0.6	Ti	0.8	0.3
Zr	1	0.2	Zn	14.9	4.8	Zn	13.8	4.2
			Zr	0.9	0.2	Zr	0.8	0.2
**5**			**6**			**7**		
**Element**	**%weight**	**%atom.**	**Element**	**%weight**	**%atom.**	**Element**	**%weight**	**%atom.**
C	2.6	4.6	C	5.4	9.4	O	47.3	64.2
O	47.9	63.3	O	46.7	60.6	F	2.3	2.6
F	0.9	1	F	1.1	1.2	Na	11.2	10.6
Na	11.2	10.3	Na	10.6	9.6	Al	0.4	0.3
Al	0.4	0.3	Al	0.3	0.2	Si	21.4	16.5
Si	19.8	14.9	Si	17.9	13.2	K	0.2	0.1
Ti	0.6	0.3	K	0.2	0.1	Ti	0.7	0.3
Zn	15.6	5.1	Ti	0.5	0.2	Zn	15.4	5.1
Zr	1	0.2	Fe	0.4	0.1	Zr	1.2	0.3
			Zn	16.2	5.1			
			Zr	0.8	0.2			

**Table 4:** Main material components identified in the ceramic coatings and their expected functional role in optical and photovoltaic performance.

Component Class	Representative Species	Main Function in the Coating	Expected Impact on Optical and PV Behavior
Glass–frit matrix	SiO_2_, Zn-containing phases	Forms the vitrified ceramic matrix, promotes adhesion to glass, and stabilizes the coating during firing	Provides the structural optical medium of the coating; depending on composition and thickness, may support relatively good transmission, especially when pigment loading is low.
Opacifying/scattering additive	Ti-containing compounds, e.g., TiO_2_	Increases scattering, opacity, and color modulation	Reduces transmitted photon flux mainly through scattering and partial reflection, which can lower $I_{SC}$ and consequently $P_{max}$.
Color-forming pigments	Fe-containing oxides, Co-containing species	Produces decorative tint and wavelength-selective absorption in the visible range	Alters spectral transmission and typically lowers $I_{SC}$ by increasing visible-light absorption; the effect on $V_{OC}$ is expected to be minor compared with the effect on current.
Minor stabilizing additives	Al-containing compounds, e.g., Al_2_O_3_; F-containing species; Zr-containing compounds	Improves chemical durability, thermal stability, and processing behavior	Usually has no dominant direct effect on PV output on its own, but can indirectly influence long-term optical stability and coating performance.

## Data Availability

The original contributions presented in this study are included in the article. Further inquiries can be directed to the corresponding authors.
